# The correlation study between *TOP2A* gene expression in circulating tumor cells and chemotherapeutic drug resistance of patients with breast cancer

**DOI:** 10.1007/s12282-024-01553-x

**Published:** 2024-04-01

**Authors:** Jin-hui Ye, Jian Yu, Ming-ying Huang, Yue-mei Mo

**Affiliations:** Department of Breast Disease, The First People’s Hospital of Zhaoqing, Zhaoqing, 526021 Guangdong China

**Keywords:** Breast cancer, Circulating tumor cell, RNA in situ hybridization, Progression-free survival

## Abstract

**Background:**

Patients with breast cancer (BC) at advanced stages have poor outcomes because of high rate of recurrence and metastasis. Biomarkers for predicting prognosis remain to be explored. This study aimed to evaluate the relationships between circulating tumor cells (CTCs) and outcomes of BC patients.

**Patients and methods:**

A total of 50 female were enrolled in this study. Their diagnoses were determined by clinical characteristics, image data, and clinical pathology. CTC subtypes and *TOP2A* gene expression on CTCs were detected by CanPatrol™ technology and triple color in situ RNA hybridization (RNA-ISH), which divided into epithelial CTCs (eCTCs), mesenchymal CTCs (MCTCs), and hybrid CTCs (HCTCs) based on their surface markers. Hormone receptor, including estrogen receptor (ER), progesterone receptor (PR), and human epidermal growth factor receptor 2 (HER-2) expression, was measured by immunohistochemistry (IHC) method before treatment. The risk factors for predicting recurrence and metastasis were calculated by COX risk regression model. The progression-free survival (PFS) of patients was determined using Kaplan–Meier survival curve.

**Results:**

The patients with a large tumor size (≥ 3 cm) and advanced tumor node metastasis (TNM) stages had high total CTCs (TCTCs) (*P* < 0.05). These patients also had high *TOP2A* expression level. COX risk regression analysis indicated that *TOP2A* expression levels in TCTCs, ER + , HER-2 + , and TNM stages were critical risk factors for recurrence and metastasis of patients (*P* < 0.05). The PFS of patients with ≥ 5 TCTCs, ≥ 3 HCTCs, and positive *TOP2A* expression in ≥ 3 TCTCs was significantly longer than that in patient with < 5 TCTCs, < 3 HCTCs, and *TOP2A* expression in < 3 TCTCs (*P* < 0.05). In contrast, the PFS of patients with positive hormone receptors (ER + , PR + , HER-2 +) also was dramatically lived longer than that in patients with negative hormone receptor expression.

**Conclusions:**

High TCTC, HCTCs, and positive *TOP2A* gene expression on CTCs were critical biomarkers for predicting outcomes of BC patients. Positive hormone receptor expression in BC patients has significant favor PFS.

## Introduction

Globally, breast cancer (BC) is one of the most lethal cancers. Its mortality rate ranks the seconding leading cause of cancer death after lung cancer in women, which account for ~ 30% in cancers of female patients [1, 2]. According to the World Health Organization (WHO) data, an estimated 2 million new cases of BC are diagnosed each year over the world, and the incidence of BC is increasing annually due to factors, such as increased life expectancy, lifestyles, genetic background, and medicine conditions [3–5]. The incidence of BC varies depending on geographical location, age, and ethnical religion. Typically, high rates of BC are seen in developed countries and in women beyond the age of 50 years old.

Currently, treatments of BC patients include surgery, chemotherapy, hormonal therapy, targeted therapy, immune therapy, and radiation therapy [6–9]. Among these therapeutic methods, chemotherapy is a major tool in patients with advanced stages. However, most patients have eventually developed drug resistance because of various mechanisms. The studies indicated that *Top2A* gene plays a critical role in drug resistance development, which encode DNA topoisomerase IIα gene [10, 11]. Topoisomerase inhibitor like doxorubicin was extensively used for chemotherapies of cancer patients [10]. However, the studies indicated that high topoisomerase IIα level is strongly associated with drug resistance of doxorubicin treatment [11–13]. Therefore, *Top2A* gene expression level is dramatically relevant to therapeutic efficiency and prognosis of chemotherapy.

Cancer cells can travel from primary tumor and invaded adjacent tissues to other parts of the body through the bloodstream or lymphatic system. This is major mechanism for cancer metastasis, which is complex and remains defined. Recent studies showed that circulating tumor cells (CTCs) in peripheral blood are significantly associated with relapse, metastasis, and prognosis of cancer [14–16]. The presence and the number of CTCs have been shown to be associated with a poorer prognosis in various types of cancers, including breast, prostate, colorectal, and lung cancer [17–20]. CTCs are thought to play a role in the spread of cancer throughout the body, and their presence in the bloodstream can be an indicator of the potential for metastasis. During this process, CTCs experience an epithelial–mesenchymal transition (EMT) mechanism with epithelial markers loss and increased expression of mesenchymal genes [21, 22]. CTCs can be divided into epithelial CTCs (ECTCs) with positive *EpCAM* and *CK8/18/19* gene expression, mesenchymal CTCs (MCTCs) with positive *Vimentin* and *Twist* genes expression, and hybrid CTCs (HCTCs) with both lineage markers expression [23, 24]. In addition, studies have indicated that patients with higher numbers of CTCs tend to have a poorer response to treatment and a shorter overall survival (OS) compared to those with lower number of CTCs [25, 26].

Therefore, monitoring CTC levels can be useful in assessing the prognosis of cancer patients and guiding treatment decision. In addition, the clinical significances of *TOP2a* gene expression in CTCs of BC patients are unclear. Here, we hypothesized that *TOP2a* gene expressive level on CTCs of BC patients is strongly relative to chemotherapeutic efficiency and the prognosis. To address this hypothesis, we employed TRIRNA and IHC techniques to measure *TOP2a* gene on CTCs and HR protein expressions of BC patients. Then we calculated PFS of patients with differentiated *TOP2a* gene and HR protein expressions. Therefore, the purpose of this study aimed to validate relationships between *TOP2a* gene expression on CTCs and outcomes of BC patients.

## Patients and methods

### Patients

A total of 50 BC patients were recruited in this study. All patients were female with 31–73-year-old age. These patients were admitted to the First People’s Hospital of Zhaoqing from 4 June, 2020 to 21 Aug, 2021. Inclusion criteria for this study were following: (I) age was more than 18 years old; (II) BC diagnosis was confirmed with combination of clinical symptoms, computerized tomography (CT) scan, ultrasound, and tumor biopsy or fine-needle aspiration cytology by clinical pathologist; (III) The tumor-node-metastasis (TNM) stages were obtained following the diagnosis criteria by AACR-8th edition [27]; (IV) estrogen receptor (ER), progesterone receptor (PR), and epidermal growth factor receptor 2 (HER2) expression were detected by immunohistochemistry (IHC) method before treatment; (V) CTCs number/5 ml (mL) were measured CanPatrol™ technology and three-color RNA-ISH before treatments; (VI) all cases were primary breast cancer. Exclusion criteria were following: (I) metastatic breast cancer from other organs; (II) follow-up loss; (III) incomplete clinical, laboratory, and images data; (IV) underwent any kinds of treatment like surgery, chemotherapy, and radiotherapy.

### Blood collecting and cell enrichment

A total of 5 mL peripheral blood from enrolled patients was drawn and immediately transferred into an ethylene-diamine-tetra-acetic acid (*EDTA*) coated tube one day just before treatment, which stored at 4 °C and processed for the next step in 4 h (h). Previous peripheral blood was mixed with 15 mL red blood lysis buffer for 30 min (min) at room temperature (RT) and spin 5 min at 1500 revolutions per minutes (RPM). Then the supernatant was discarded and washed twice with phosphate-buffered saline (PBS). The collected cells were fixed with 4% paraformaldehyde (PFA) for 8 min and connected with the vacuum filtration system at 0.08 MPa following described method in the literature [28].

### Identification of CTCs by multiple gene mRNA probes using RNA in situ hybridization (RNA-ISH)

To get CTCs lineage specific and *TOP2A* gene expression on CTCs, the above fixed cells were treated with 0.1% (mg/mL) proteinase K for 30 min at 4 °C to enhance the cell membrane permeability of targeting gene probes. After washing twice with PBS solution, a serial of lineage specific capture probes, including epithelial biomarker probes: *EpCAM* and *CK8/18/19*; mesenchymal biomarker probes: *Vimentin* and *Twist*; and *TOP2A* mRNA probe, were added hybridization solution and incubated for 2 h at 40 °C. To remove unbound probes, cells were washed twice with 0.1 × SSC eluent (Sigma, St. Louis, USA). Then cells were incubated for 90 min at 40 °C with the pre-amplification and the amplification solution to amplify targeting genes signal. Finally, three specific fluorescence conjugated gene mixtures, including Alexa Fluor (AF) 594 for probe EpCAM and CK8/18/19; AF488 for probe Vimentin and Twist; and AF750 for *TOP2A*, were added into cells and incubated for 60 min at 40 °C. To get cell nucleus images, we also added specific staining dye6-diamidino-2-phenylindole (DAPI) into samples. All specific gene expression images were taken pictures and counted their expression levels in fluorescence scanning microscope by 100 magnification (Olympus BX53, Tokyo, Japan).

### Positive CTCs determination

**Fig. 1:**
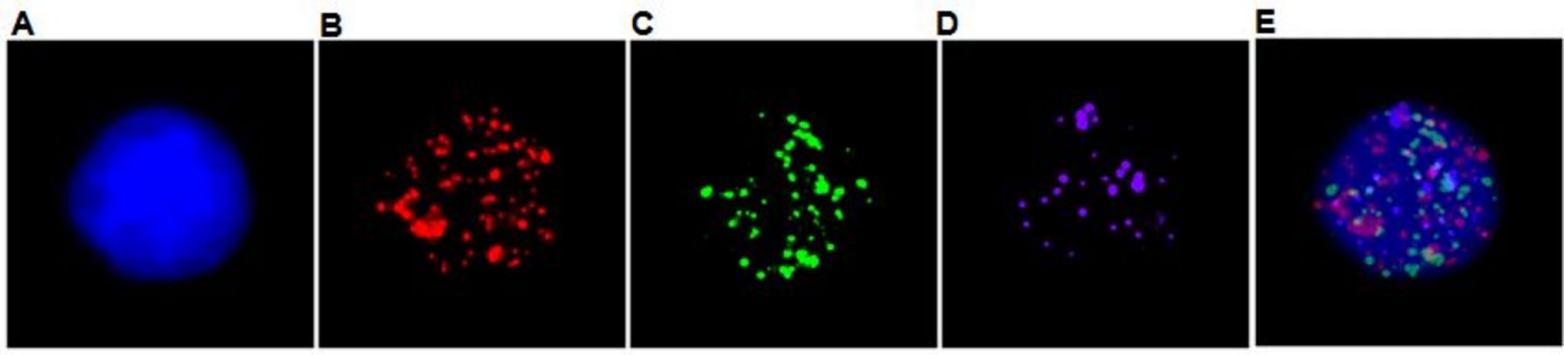
The *TOP2A* mRNA expression in CTCs. Fluorescence microscope images of different subtypes of CTCs. Red fluorescence: epithelial marker expression signal points; Green fluorescence: mesenchymal marker expression signal points; Purple fluorescence: *TOP2A* gene expression signal points; Blue fluorescence: DAPI nucleus. **A** DAPI stain nucleus; **B** epithelial type CTCs (eCTCs); **C** mesenchymal type CTCs (MCTCs); **D** *TOP2A*-positive CTCs; **E** hybrid CTCs. CTC, circulating tumor cell; DAPI, 6-diamidino-2-phenylindole

Following reagent supplier description ( SurExam, Guangzhou, China), the red dots, green dots, red and green mixed dots, purple dots stand for epithelial (eCTCs), mesenchymal CTCs (MCTCs), hybrid CTCs (hCTCs), and *TOP2A* gene expression in fluorescence microscope by 100 × magnification, respectively. Then we randomly counted 5 fields and averaged each type CTCs number. Their identification criteria and images show in Table 1 and Fig. 1.

**Table 1:** Circulating tumor cells (CTCs) Characterization

Type	Spot color	DAPI
eCTCs mCTCs hCTCs TOP2A +	Red Green Red and Green Purple	+ + + +

*eCTCs* epithelial CTCs, *mCTCs* mesenchymal CTCs, *hCTCs* hybrid CTCs, *TOP2A + * TOP2A-positive in different type CTCs, *DAPI* 6-diamidino-2-phenylindole for cell nucleus

### Hormone receptor expression using immunohistochemistry (IHC)

Hormone receptor expression levels of BC patients are closely associated with therapeutic effects and prognosis. To evaluate hormone receptor expression in BC patients, we employed IHC method to detect their levels, including ER, PR, and HER2. Briefly, the deparaffinized sections of tumor tissue from biopsy or surgery were cut into 4-μM wide and mounted on slides. The primary and secondary antibodies were incubated following the manufacturer recommended protocol (Roche Diagnostics, Basel, Switzerland). Positive ER, PR, and HER-2 cells were determined by at least three certificated pathologists following the 2018 ASCO/CAP recommended criteria [29].

### The disease status and follow-up of patients

To assess the prognostic effects of multiple variants on BC patients, we decided to follow-up to 2 years after treatment at the end on 21 Aug, 2021. All patients were met at outpatient or phone call by every three months in the first half year, then every six months in the remaining time. The follow-up information included disease symptoms, chest computed tomography (CT), whole-body bone scan, and positron emission tomography (PET-CT) scan. The criteria for recurrence and metastasis were defined as image detections showing space-occupying lesions in chest and other organs of human body. Progression-free survival (PFS) was time from treatment to recurrence.

### Statistical analysis

All data analyses were performed using GraphPad Prism 9.0 version (San Diego, California, USA). Comparison of continuous variables between CTCs levels and clinical–pathological characteristics was performed using Student *T* test, Mann–Whitney U test, and ANOVA test. In contrast, categorical variables were compared using *χ*2 tests. The prognostic factors for predicting outcome of patients were determined using univariate and multivariate Cox’s regression model. PFS comparison of different patient groups was calculated using the Kaplan–Meier curve. *P* < 0.05 was considered as significant differences.

## Results

### The clinico-pathological characteristics of patients.

A total of 50 BC patients all were female with 31–73-year-old age (median was 49-year-old, mean ± Sd was 50.38 ± 8.68-year-old). The most patients were invasive ductal carcinoma (IDC) (47 cases, 94%). The other three patients were invasive lobular carcinoma (ILC, 1 case, 2%), ductal carcinoma in situ (DCIS, 1 case, 2%), and mucinous carcinoma (MC, 1 case, 2%), respectively. There were 13 stage I, 28 stage II, 8 stage III, and 1 DCIS patients according to staging criteria recommended by AACR-8th edition [27]. We also performed estrogen receptor (ER), progesterone receptor (PR), and HER2 levels detection by IHC and found that there were 36 ER + , 28PR + , 29 HER2 + , and 6 triple-negative cases, respectively (Table 2).

**Table 2:** The characteristics and prevalence of circulating tumor cells in patients with breast cancer (*n* = 50)

	TCTCs		P	HCTCS		P	MCTCs		P	TOP2A^+^TCTCs		P
Characteristics (Case no)	≥ 5	< 5		≥ 1	< 1		≥ 1	< 1		≥ 3	< 3	
Age (years)												
≥ 60(7)	4	3	0.842	3	4	0.104	0	7	0.502	4	3	0.788
< 60(43)	18	25		26	17		2	43		21	22	
Tumor position												
Right(24)	12	12	0.125	15	9	0.905	1	23	0.741	17	6	0.063
Left(24)	11	13		10	14		1	23		10	14	
Both side (2)	0	2		1	1		0	2		0	2	
Tumor size (cm)												
≥ 3 (25)	18	7	0.029*	8	11	0.119	3	16	0.051	14	5	0.021*
< 3(25)	6	19		22	9		0	31		9	22	
Pathologic classification												
Invasive (48)	23	25	0.167	27	23	0.330	3	45	0.228	24	24	0.120
Non-invasive (2)	2	0		2	0		0	2		2	0	
Tumor stage												
I (13)	10	3	0.057	10	3	0.057	2	11	0.386	2	11	0.012*
II(29)	15	14		15	14		0	29		20	9	
III (8)	8	0		8	0		0	8		7	1	
Lymph node metastasis												
None (26)	16	11	0.117	18	9	0.079	1	26	0.732	18	9	0.135
1–3 (19)	5	13		7	11		2	16		5	13	
≥ 4(5)	4	1		5	0		0	5		4	1	
ER												
Negative (14)	10	4	0.167	11	3	0.121	0	14	0.376	9	4	0.206
Positive (36)	12	24		19	17		3	33		18	19	
PR												
Negative (20)	11	9	0.842	12	8	0.956	1	19	0.779	11	9	0.942
Positive (30)	15	15		17	13		1	28		16	14	
HER2												
Negative (22)	14	8	0.298	15	7	0.491	1	20	0.691	14	8	0.546
Positive (16)	5	11		9	7		1	15		7	9	
Borderline (13)	7	6		5	7		0	13		7	5	
Molecular type												
luminal A (8)	5	3	0.042*	7	1	0.074	0	8	0.822	5	3	0.085
luminal B (16)	8	8		7	11		1	16		8	9	
HER2-positive (19)	5	14		9	10		1	17		7	11	
Triple-negative (7)	7	0		7	0		0	7		7	0	

*TCTCs* total circulating tumor cells, *HCTCs*,hybrid circulating tumor cells, *MCTCs* mesenchymal circulating tumor cells, *ER* estrogen receptor, *PR* progesterone receptor (PR), *HER2* human epidermal growth factor receptor 2

### Comparison of CTCs number in breast cancer subgroups

To investigate total CTCs, subtypes, and *TOP2A* levels in different characteristic groups, we performed ECTC, MCTCs, hybrids CTCs (HCTCs), and *TOP2A* CTCs based on criteria in Table 1 and Fig. 1, which outlined nucleus shape with DAPI staining (Fig. 1A) and occupied red color (Fig. 1B), green color (Fig. 1C), purple color (Fig. D, *TOP2A* gene), and mixed color (Fig. 1E) expression in fluorescence microscope by 100 × magnification, respectively. We also compared differences between high CTCs and low CTCs by Mann–Whitney U test using different CTC cut-off values. The results show in Table 2. We found that there were more total CTCs when tumor size was large (≥ 3 cm). In contrast, if tumor size was < 3 cm, total CTCs number was significantly decreased (*P* = 0.029). Interestingly, this result was consistent with *TOP2A* levels. It means that when large tumor size was ≥ 3 cm and there were high total CTCs, *TOP2A* levels were greatly increased compared to small tumor size and low total CTCs (*P* = 0.021). In addition, we also found that total CTCs levels also were strongly relevant to molecular type (*p* = 0.042). Among 7 patients with triple-negative, all cases had more total CTCs. Except these obvious findings, we also identified that *TOP2A* levels were dramatically associated with TNM stages, which were significantly higher than that in patients with stage I (*P* = 0.012). These results indicated that total CTC numbers were greatly associated with tumor size and molecular type. *TOP2A* levels were closely relevant to tumor size and tumor TNM stages.

### COX regression analysis of univariate and multivariate in defined groups

To trace the relationships between the outcomes and specific clinical characteristics, we carried out COX regression analysis for patient survival using univariate, such as age, TNM stage, ER, PR, HER-2, total CTCs counts, hybrids CTCs number, and *TOP2A* level. We followed up to 24 months and recorded recurrence, metastasis, and PFS. The results show in Table 3. We found that age, TCTCs, HCTCs, and PR-positive expression were not risk factors for recurrence and metastasis in BC patients. In contrast, *TOP2A* expression in high TCTCs, ER-positive, HER-2 positive expression, and tumor staging were critical risk factors. Their hazard ratios (HR) for recurrence and metastasis in positive patients were significant high than that in negative patients (*P* < 0.05).

**Table 3:** Multivariate analysis of risk factors for recurrence and metastasis in breast cancer patients

Variate	HR	95% CI	P value
Age	1.069	0.9922 -1.166	0.1283
TCTC	7.487	0.3753-116.9	0.2089
HCTC	0.053	0.003--0.604	0.0679
*TOP2A* in CTC	0.007	0.001-0.139	0.0013
ER+	7.11	0.69-1.58	0.0192
PR+	37.91	0.263-6.771	0.1479
HER-2+	12.76	1.527 to 147.1	0.0260
TNM stages	0.001	0.007-0.037	0.0021

*TCTC* total circulating tumor cells, *HCTC* hybrid circulating tumor cells, *ER* positive estrogen receptor, *PR* progesterone receptor, *HER-2* human epithelial growth factor receptor2, +, positive, *TNM* tumor-node-metastasis, *HR* hazard ratio, *CI* confidence interval.

### Kaplan–Meier survival analysis in patients with various CTCs and hormone receptor expression

**Fig. 2:**
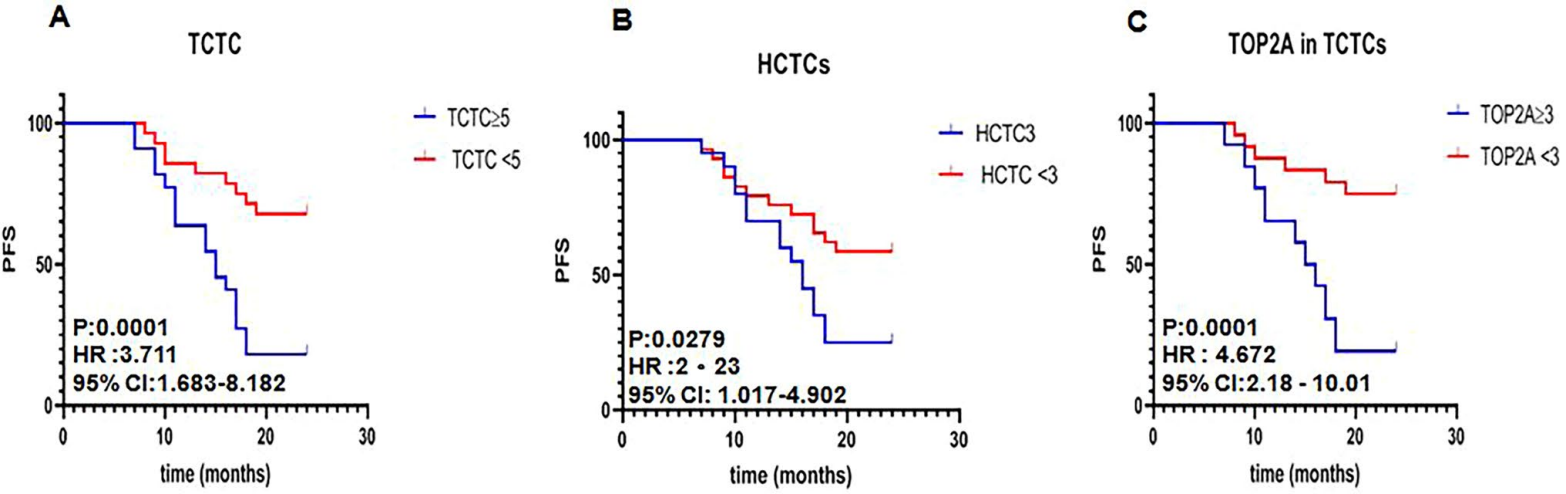
Survival curve of CTCs and TOP2A in TCTCs. **A** PFS Comparison between patients with ≥ 5 TCTCs and < 5 TCTCs; **B** PFS Comparison between patients with ≥ 3HCTCs and < 3 HCTCs; **C** PFS Comparison between patients with ≥ TOP2A in ≥ 3 TCTCs and < 3 TCTCs; *TCTCs* total circulating tumor cells, *HCTCs* hybrid circulating tumor cells, *PFS* progression-free survival, *HR* hazard ratio, *CI* confidence interval

To evaluate outcomes of patients with different CTCs, *TOP2A*, and hormone receptors, we performed survival analysis using Kaplan–Meier survival curve. The results showed in Fig. 2. We compared PFS of patients with ≥ 5 TCTCs and < 5 TCTCs (Fig. 2A), ≥ 3 HCTCs and < 3 HCTCs (Fig. 2B), and *TOP2A* in ≥ 3 TCTCs and < 3 TCTCs (Fig. 2C). It was found that PFS of patients with low TCTCs, HCTCs, and *TOP2A* was significant longer that in patients with high TCTCs, HCTCs, and TOP2A. Their hazard ratios (HR), 95% confidence interval (CI), and P value were HR 3.711, 95% CI 1.683–8.182, *P* = 0.0001 for TCTCs; HR 2.23, 95% CI 1.017–4.902, *P* = 0.0279 for HCTCs; HR 3.711,95% CI 1.683–8.182, *P* = 0.0001 for TCTCs; HR 4.672, 95% CI 2.28–10.01, *P* = 0.0001 for TOP2A in TCTCs (Table 4). These results indicated that high CTCs and *TOP2A* are critical factors for the outcomes of breast cancer patients.

**Table 4:** Survival analysis of different risk factors in breast cancer patients

Variate	HR	95% CI	P value
TCTC	3.711	1.683 - 8.182	0.0001
HCTC	2.23	1.017-4.902	0.0279
*TOP2A* in TCTC	4.672	2.28-10.01	0.0001
ER+	3.207	0.1258-0.8175	0.0028
PR+	0.4324	0.1992-0.9383	0.0411
HER-2+	0.4724	0.1942 -0.9681	0.0414

*TCTC* total circulating tumor cells, *HCTC* hybrid circulating tumor cells, *ER* positive estrogen receptor, *PR* progesterone receptor, *HER-2* human epithelial growth factor receptor2, +, positive, *HR* hazard ratio, *CI* confidence interval.

**Fig. 3:**
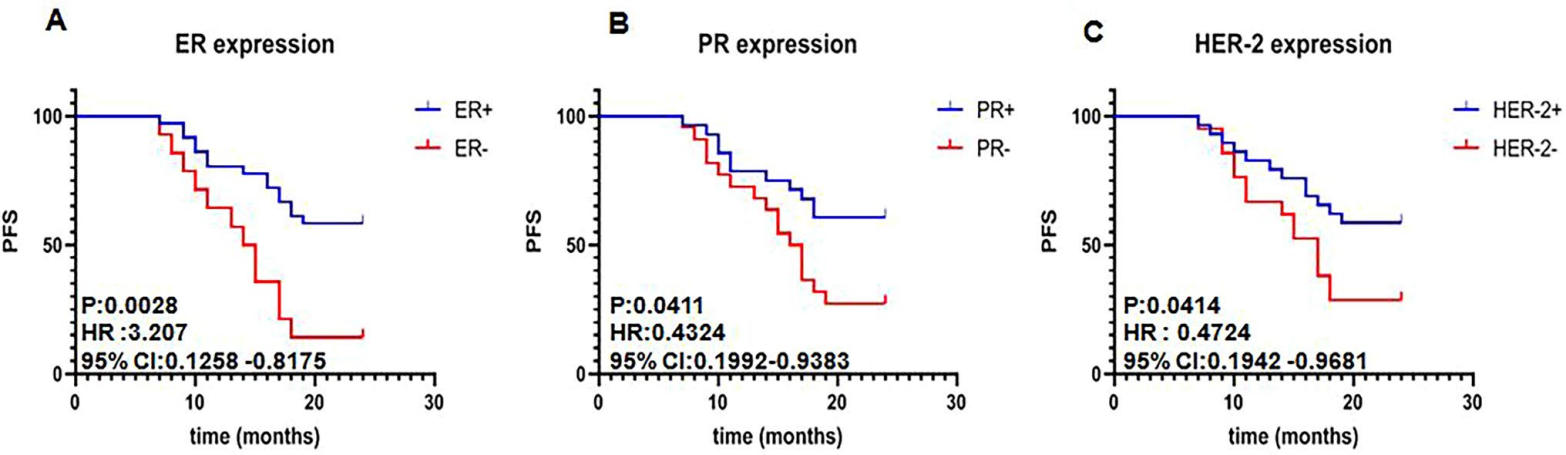
Survival curve of breast cancer patient with different hormone receptor. **A** PFS Comparison between patients with ER-positive and ER-negative; **B** PFS Comparison between patients with PR-positive and PR-negative; **C** PFS Comparison between patients with HER-2 positive and HER-2 negative. *PFS* progression-free survival, *HR* hazard ratio, *CI* confidence interval

We also compared the PFS of BC patients with ER, PR, HER-2-positive and -negative patients. The results show in Fig. 3 and Table 4. We found that the PFS of patients with positive ER, PR, and HER-2 was dramatically longer free disease status than that in patients with negative hormone receptors. Their statistical data were HR 3.207, 95% CI 0.1258–0.8172, *P* = 0.0028 for ER + patients; HR 0.4324, 95% CI 0.1992–0.9383, *P* = 0.0411 for PR + patients; HR 0.4737, 95% CI 0.1942–0.9681, *P* = 0.0414 for HER-2 + patients. These results revealed that positive hormone receptor expression in BC patients has favor to their prognosis.

## Discussion

The present data indicate the relevance of TCTCs, HCTCs, and *TOP2A* on TCTCs are critical biomarkers for predicting outcomes of BC patients. Positive ER, PR, and HER-2 expressions also have favorable BC patients survival. CanPatrol^TM^technology and RNA in situ hybridization (RNA-ISH) are sensitive and reliable methods for CTC detection.

Breast cancer is an extensive prevalent disease in women and has increasing incidence in recent years [30]. Chemotherapy is a major tool for patients with advanced stages and adjuvant treatment after surgery. However, the most BC patients eventually became recurrence and metastasis because of chemotherapy drug resistance. Therefore, it is urgent to identify a sensitive and reliable technology for predicting the outcomes of BC patients. Recently, CTC detection for predicting the recurrence of cancer was extensively reported [28, 31–33]. Li et al. [28] reported that CTCs measurement in non-small cell lung cancer (NSCLC) using CanPatrol technology and RNA-ISH can distinguish benign and carcinoma patients at 0.5 CTCs/5 mL cut-off of peripheral blood and obtained 81.6% sensitivity and 86.8% specificity. This result revealed that CanPatrol combined RNA-ISH can test rare CTCs using a little volume blood. Dong et al. [33] also used this method to determine CTCs and PD-L1 expression. They found that high TCTCs, MCTCs, and PD-L1 ( +) CTCs had poor prognosis in NSCLC patients. Here, our results confirmed that CanPatrol combined RNA-ISH can detect rare CTCs and *TOP2A* gene in 5 mL peripheral blood. Our TCTCs and HCTCs count in BC patients had poor prognosis and was consistent with previous report [32].

*TOP2A* gene was found to be involved in many chemotherapeutic drug resistances because it engages DNA replication, repair, recombination and transcription [10, 11, 34]. *TOP2A* gene is highly expressed in proliferative cells and clue that it is involved in aggressive invasive behaviors of cancer cells [35]. Yang et al. [36] and Menendez et al. [37] investigated *TOP2A* gene expression in breast cancer MCF-7 cell line or SK-Br3 cell line and found that wild type MCF-7 cell and SK-Br3 cells had high *TOP2A* gene expression. *TOP2A* inhibitor administration can induce MCF-7 cell and SK-Br3 cells apoptosis. Here, our data show that *TOP2A* is not only a risk factor for recurrence and metastasis of BC patients, but also a key biomarker for predicting the prognosis of BC patients. We found that *TOP2A*-positive expression in ≥ 3 TCTCs had dramatically affected the PFS of patients. This result further provided a new mechanism for drug resistance.

Previous studies indicated that ER, PR, HER-2 expressions in BC patients were closely associated with treatment of patients [38, 39]. Indeed, our current data confirmed that ER + , PR + , HER-2 + expression had favorable prognosis although PR + was not relevant to risk factor for recurrence and metastasis. There results confirmed previous reports [31, 32].

**Limitations:** This study has interesting findings. However, there are a few limitations: (I) Total sample size is 50 patients. This sample size is limited and may product some sample bias; (II) Present results were from a single of clinical center, which may have a selective bias; (III) There are limited mechanism studies between CTCs number and BC tumorigenesis; (IV) *TOP2A* expression is how to be involved in the outcomes of BC patients. To overcome these limitations, we will recruit more patients from multiple hospitals and perform deep biological mechanism study in the further.

## Data Availability

The datasets generated and analyzed are available from the corresponding author upon reasonable request.
